# Potential Importance of Early Focal Radiotherapy Following Gross Total Resection for Long-Term Survival in Children With Embryonal Tumors With Multilayered Rosettes

**DOI:** 10.3389/fonc.2020.584681

**Published:** 2020-12-17

**Authors:** Lisa Mayr, Johannes Gojo, Andreas Peyrl, Amedeo A. Azizi, Natalia M. Stepien, Thomas Pletschko, Thomas Czech, Christian Dorfer, Sander Lambo, Karin Dieckmann, Christine Haberler, Marcel Kool, Irene Slavc

**Affiliations:** ^1^ Department of Pediatrics and Adolescent Medicine and Comprehensive Center for Pediatrics, Medical University of Vienna, Vienna, Austria; ^2^ Department of Neurosurgery, Medical University of Vienna, Vienna, Austria; ^3^ Division of Pediatric Neurooncology, Hopp Children’s Cancer Center Heidelberg (KiTZ), Heidelberg, Germany; ^4^ Division of Pediatric Neurooncology, German Cancer Research Center (DKFZ) and German Cancer Consortium (DKTK), Heidelberg, Germany; ^5^ Department of Radiotherapy, Medical University of Vienna, Vienna, Austria; ^6^ Division of Neuropathology and Neurochemistry, Department of Neurology, Medical University of Vienna, Vienna, Austria; ^7^ Research Department, Princess Máxima Center for Pediatric Oncology, Utrecht, Netherlands

**Keywords:** embryonal tumor with multilayered rosette, embryonal tumor with abundant neuropil and true rosette, radiotherapy, focal radiotherapy, intrathecal therapy, embryonal brain tumors

## Abstract

Embryonal tumor with multilayered rosettes (ETMR) is a rare, aggressive embryonal central nervous system tumor characterized by LIN28A expression and alterations in the *C19MC* locus. ETMRs predominantly occur in young children, have a dismal prognosis, and no definitive treatment guidelines have been established. We report on nine consecutive patients and review the role of initiation/timing of radiotherapy on survival. Between 2006 and 2018, nine patients were diagnosed with ETMR. Diagnosis was confirmed histopathologically, immunohistochemically and molecularly. Median age was 25 months (5–38). Location was supratentorial in five, pineal in three, and brainstem in one. Seven patients had a gross total resection, one a partial resection and one a biopsy at initial diagnosis. Chemotherapy augmented with intrathecal therapy started a median of 10 days (7–20) after surgery. Only two patients who after gross total resection received radiotherapy very early on (six weeks after diagnosis) are alive and in complete remission 56 and 50 months after diagnosis. All remaining patients for whom radiotherapy was deferred until the end of chemotherapy recurred, albeit none with leptomeningeal disease. A literature research identified 228 patients with ETMR. Including our patients only 26 (11%) of 237 patients survived >36 months with no evidence of disease at last follow-up. All but two long-term (>36 months) survivors received radiotherapy, ten of whom early on following gross total resection (GTR). GTR followed by early focal radiotherapy and intrathecal therapy to prevent leptomeningeal disease are potentially important to improve survival of ETMR in the absence of effective targeted therapies.

## Introduction

Embryonal tumors with multilayered rosettes (ETMR) are rare and highly aggressive embryonal brain tumors primarily affecting infants and young children under the age of four years. ETMRs encompass three histological variants: embryonal tumor with abundant neuropil and true rosettes (ETANTR), ependymoblastoma, and medulloepithelioma (1, 2), all of which were previously regarded as subtypes of central nervous system (CNS) primitive neuroectodermal tumors (PNETs). Histologically, the presence of undifferentiated neuroepithelial cells forming multi-layered rosettes is characteristic of this tumor entity. Broad bands of well-differentiated neuropil islands, ependymoblastic rosettes, or medulloepitheliomatous rosettes may be present (3). Furthermore, they are typically characterized by a focal amplification spanning the *C19MC* microRNA cluster at 19q13.42 and high expression of the stem cell marker LIN28A (4). Based on this sensitive and specific marker irrespective of other features, Paulus and Kleihues proposed the term “Embryonal tumors with multilayered rosettes” as unifying entity for these tumors (5). In the updated version of the World Health Organisation (WHO) Classification of Tumours of the Central Nervous System, ETMRs are therefore defined as aggressive CNS embryonal tumors with multi-layered rosettes and alterations in the *C19MC* locus at 19q13.42, *i.e.* ETMR, *C19MC*-altered, grade IV. Cases of embryonal tumors with multi-layered rosettes without alteration in the *C19MC* locus or that were not tested for alteration are classified as ETMR, not otherwise specified (NOS) (6). ETMR may originate anywhere in the brain with approximately two thirds occurring in the cerebral hemispheres and one third in the brain stem and cerebellum (7). Neuroimaging usually reveals large, solid masses featuring irregular- or no-contrast enhancement, often with significant mass effect. Some cases show cystic components and microcalcifications (8, 9). ETMRs may present with or develop leptomeningeal dissemination as well as extracranial invasive growth in the soft tissues and extracranial metastases. Patients with ETMR have a dismal prognosis with reported survival times averaging 12 months despite maximal safe resection followed by intensive multimodal therapy often including high-dose chemotherapy (HDCT) and delayed radiotherapy (8). Due to the rarity of the disease fewer than 300 cases have been reported so far. Given the young age of the patients, radiotherapy was often deferred and no definitive guidelines for optimal treatment have been established.

We report on the importance of an early focal radiotherapy on the outcome in a series of nine consecutive patients with ETMR, treated at the Medical University of Vienna (MUV) since 2006.

## Methods

Reassessment of all highly malignant embryonal brain tumors formerly classified as ETANTR, medulloepithelioma, ependymoblastoma, or CNS PNET with regard to LIN28A expression and amplification of the *C19MC* microRNA cluster disclosed nine patients treated at the MUV since 2006.

Seven patients previously diagnosed as ETANTR or ETMR were reclassified as ETMR. Two patients (cases 1 and 3) originally diagnosed as CNS PNET were reclassified. Male to female ratio was 2:7. Median age at diagnosis was 25 months (range 5–38). Five tumors were located supratentorially, three in the pineal region and one in the pons. At presentation no patient showed signs of leptomeningeal dissemination. The extent of surgical resection was defined on postoperative magnetic resonance imaging (MRI) performed within 48 hours as gross total resection (GTR, no obvious residual tumor), subtotal resection (STR with <10% residual tumor), partial resection (PR with 10–50% residual tumor) and biopsy (>50% residual tumor). Determination of progression or response was based on response assessment in neuro-oncology (RANO) criteria (10). Clinical characteristics are depicted in **Table 1**. Informed consent was obtained for all patients.

**Table 1:** Patient characteristics, treatment and outcome.

Case	Age (months)	Sex	Location	Surgery	M-Stage	Primary CT	Focal RT (months after Dx)	Intrathecal CT	HDCT (months after Dx)	Status/Follow up (months)	LIN28A	*C19MC* amplification/*DICER1* mut	CNV Gains	CNV Losses
1	25	m	parietal	GTR	M0	HIT 2000 SKK	11.5	VP16, Depocyte MTX,	6	DOD,27	pos	19q13.42	2,5p	–
2	33	f	parietal	GTR	M0	HIT2000 SKK	7.5	VP16, Depocyte	6	DOD,14	pos	19q13.42	1q,2,4,17,20	–
3	5	f	pineal	GTR	M0	HIT2000 SKK	–	VP16, Depocyte	6	DOC,6	pos	DICER1	2	7q
4	35	f	bifrontal	GTR	M0	Doxorubicin	–	no	–	DOD,15	pos	19q13.42	2,3,4,8,11,12	–
5	16	f	pineal	GTR	M0	MUV ATRT	6.5	VP16	–	DOD,13	pos	19q13.42	–	–
6	38	f	parietal	GTR	M0	PEI/TMZ	1.5	VP16, Depocyte Topo	–	NED,56+	pos	19q13.42	–	–
7	27	f	bifrontal	GTR	M0	PEI/TMZ	1.5	VP16, Depocyte	–	NED,50+	pos	19q13.42	4	–
8	13	f	pontine	Biopsy	M0	IP-CZD	3.5	VP16	–	DOD,11	pos	19q13.42	2,7,8	–
9	19	m	pineal	PR	M0	IP-CZD	5.5	VP16, ara-C, Topo	–	DOD,14	pos	19q13.42	2	–

m, male; f, female; GTR, gross total resection; PR, partial resection; CT, chemotherapy; PEI, cisplatin, etoposide, ifosfamide; TMZ, temozolomide; IP-CZD, polish infant IP-CZD protocol; RT, radiotherapy; Dx, diagnosis; VP16, etoposide; Depocyte, liposomal cytarabine; MTX, methotrexate; Topo, topotecan, ara-C, aqueous cytarabine; HDCT, high-dose chemotherapy; DOD, dead of disease; DOC, dead of other cause; NED, no evidence of disease; CNV, copy number variations.

### Histopathology, Interphase Fluorescence *In Situ* Hybridization

For diagnostic purposes routine histopathological examination on formalin-fixed and paraffin embedded (FFPE) tissue was performed including immunohistochemical (IHC) analysis with an anti-LIN28A polyclonal antibody (antibody (A177 #3978, 1:800, Cell Signaling Inc; Danvers, MA 01923, USA). Interphase Flourescence In Situ Hybridization (iFISH) was carried out on FFPE tissue sections as previously described (11) using a commercially available 19q13.42 (*C19MC*, spectrum orange) and 19.13.12 (spectrum green) probe (CytoTest *C19MC*/TPM4), Diagnostic Technology Pty Ltd; Belrose, Australia).

### Methylation Array and Whole Genome Sequencing Analyses

To analyze methylation-specific subgrouping, copy number aberrations and somatic/germline mutations in more detail DNA was extracted from fresh frozen tumor tissue in all nine cases. Matched blood samples for germline sequencing were available in six cases. Methylation array (Illumina 850K platform) and whole genome sequencing (40×) were performed *via* the DKFZ core facility. All analyses were performed according to national and international biosafety guidelines and as previously published (12, 13).

### Systemic Chemotherapy

All patients started chemotherapy a median of 10 days (range 7–20 days) after surgery. Intensive chemotherapy followed various brain tumor protocols (HIT SKK (14), MUV ATRT (15), and the Polish infant protocol IP-CZD consisting of VCR 1.5 mg/m^2^, etoposide 300 mg/m^2^, cisplatin 100 mg/m^2^ alternating with cyclophosphamide 1.5 g/m^2^ per course) and included cisplatin or carboplatin and etoposide in all except one patient (hypoxic brain injury) who received doxorubicin based treatment. Three patients who were in complete remission after GTR and conventional chemotherapy received HDCT consisting of a modified “Head Start” protocol (carboplatin, etoposide and Thiotepa) (16) (**Table 1**). Chemotherapy was intended to be given for approximately six months and to be followed by HDCT in patients still in remission in order to delay radiotherapy in this vulnerable population.

Two patients aged 38 and 27 months at diagnosis (cases 6 and 7) were treated differently. Both patients started focal radiotherapy exactly six weeks after GTR. The time for planning radiotherapy (as well as second look surgery in one of the patients) was bridged by two courses of PEI (cisplatin 20 mg/m^2^ d1-5, ifosfamide 1.500 mg/m^2^ d1-5, and etoposide 100 mg/m^2^ d1-3) in both patients. Temozolomide was given concomitant to focal radiotherapy and continued thereafter for a total of 12 courses (17).

### Intrathecal Therapy

Therapy was augmented with intrathecal chemotherapy in all except one patient (hypoxic brain injury) and consisted of alternating etoposide (0.25 mg <1-year-old; 0.5 mg >-year-old on five consecutive days), and/or methotrexate (2 mg/day for four days), and/or liposomal cytarabine (Depocyte) (25 mg <3-year-old and 35 mg >3 and <9-year-old and 35–50 mg >9-year-old) in combination with oral dexamethason to prevent chemical arachnoiditis and/or topotecan (0.25 mg >1 and <2–year-old, 0.32 mg >2 and <3-year-old, and 0.4 mg >3-year-old) twice a week and/or aqueous cytarabin 30 mg twice a week (16 mg <1-year-old, 20 mg >1 and <2-year-old, 26 mg >2 and <3-year-old) (18–23).

### Radiotherapy

All except two patients (case 3 who died after bone marrow transplant and case 4 with hypoxic brain injury) received focal irradiation. Six were treated with photons and one with protons. Three dimensional (3D) treatment planning was performed in all patients. Target definitions were based on pre- and postoperative MRIs.

Gross tumor volume (GTV) included the postoperative tumor bed. Clinical target volume (CTV) was defined as GTV plus 1 cm margin adapted to the anatomical structures. Planning target volume (PTV) was CTV plus 3 mm margin. All but one patient were treated with conformal volume modulated radiotherapy (VMAT) and photons. One patient was treated with protons. Target definition was identical for protons and photons based on MRI. Fraction sizes were 1.8 Gy for all target volumes with a total dose of 54 Gy in all patients. None of the patients was reirradiated.

### Treatment at Recurrence

Besides additional resections and radiotherapy three patients received temozolomide and three patients received a metronomic antiangiogenic therapy according to MEMMAT (ClinicalTrials.gov Identifier: NCT01356290), one also topotecan and olaparib. Olaparib was given continuously at a dose of 50 mg BID (100 mg total dose per day) in this 2.4-year-old male (body weight 13 kg) and combined with topotecan given at a dose of 1.5 mg/m^2^ for five consecutive days every three weeks (**Supplementary Table S1**).

### Neuropsychological and Quality Of Life Testing

All patients were tested with age-appropriate methods. Domains tested in the two long-term survivors included fluid intelligence, verbal comprehension, processing speed, memory, working memory, attention, fine motor functions, executive functions and emotion recognition (24–33). The test-battery was accompanied by a brief behavioral screening (26, 34, 35). Additionally, patients’ health-related quality of life (HRQoL) was assessed using proxy-reports (parents) (36).

### Statistical Analysis

For clustering of the DNA methylation profiles of ETMR cases treated at MUV t-Stochastic neighborhood embedding with 193 ETMRs (12), 534 other CNS tumors (37) and 44 pineoblastomas (13) were used.

### Ethics Approval

Ethics approval and consent to participate: The ethics committee of the MUV granted their permission in 2016 with the number 1244/2016.

## Results

### Surgery

Seven patients had primary GTRs as evidenced by MRI performed within 48 h after surgery. Two patients who had an initial biopsy and PR, respectively, at another hospital and were progressive under adjuvant intensive chemotherapy had a second PR and STR, respectively, three and four months after original diagnosis (cases 8 and 9).

### Histopathology, iFISH

All tumors showed characteristic morphological features of ETMR including multi-layered rosettes and neuropil islands as well as immunohistochemical expression of LIN28A. Gain/amplification at the *C19MC* locus was detected in eight of nine tumors by iFISH.

### Methylation Array and Whole Genome Sequencing

**Figure 1:**
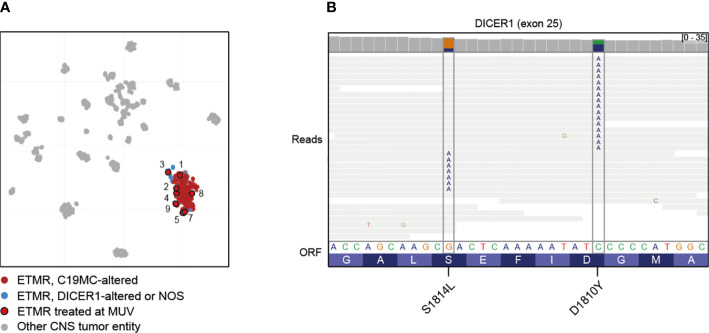
Molecular characterization of treated cases. **(A)** Clustering of ETMRs treated at MUV using t-Stochastic neighborhood embedding with 193 ETMRs (12), 534 other CNS tumors (37) and 44 pineoblastomas (13). Case 6 was not included due to low tumor content. **(B)** Mutual exclusivity of mutations affecting *DICER1* exon 25 in case 3, suggesting that the mutations are biallelic. Lines represent reads; substitutions are annotated in the reads at the appropriate position.

Methylation-based classification was performed in all nine cases (**Figure 1A**, **Supplementary Figures S1–S9**). In one case (case 6) tumor cell content was too low to allow classification by methylation array despite a clear amplification of *C19MC* confirming the diagnosis of ETMR (**Supplementary Figure S6**). In accordance to iFISH, all except one case harbored the typical *C19MC*-amplification. All tumors were further analyzed by whole genome sequencing which revealed *DICER1*-mutation in the single case without *C19MC*-amplifcation (**Supplementary Figure S3**). For this case no germline material was available to test for germline *DICER1*-mutation, but the presence of two different mutations being mutually exclusive in the reads suggested that both alleles were affected (**Figure 1B**). Further detected somatic mutations are summarized in the supplementary material (**Supplementary Figures S1–S9**). With respect to other copy number variations (CNVs) gain of chromosome 2 was most common (6/9) as previously reported by Sin-Chan et al. (38) followed by gain of chromosomes 4 (3/9) and 8 (2/9). Interestingly, we also found gain of chromosome 5p in one case (**Table 1**, **Supplementary Figure S1**).

### Response to Systemic Treatment

Only two patients who after GTR received radiotherapy very early on (six weeks after diagnosis) following two courses of PEI are alive and in complete remission 56 and 50 months after diagnosis. All remaining patients in whom radiotherapy was deferred until the end of chemotherapy recurred. In cases with an initial GTR relapse occurred after a median of six months (range 9 weeks–11 months) and the patients succumbed to their disease after a median of 14 months (**Table 1**). In the two patients with an initial biopsy and PR, respectively, the tumor progressed steadily as evidenced in six-weekly MRIs despite intensive therapy. Both received a very good PR and STR before focal radiotherapy concomitant and followed by temozolomide and both also died of tumor recurrence/progression 11 and 14 months after initial diagnosis.

### Response to Intrathecal Therapy

All except the patient with hypoxic brain injury received intrathecal therapy. Intrathecal chemotherapy consisted of etoposide in all eight patients alternating with liposomal cytarabine (Depocyte) every two weeks in five patients. Two patients received only etoposide every two weeks and one patient etoposide alternating with aqueous cytarabine on days 1, 4, 8 and 11 because production of the slow release formulation was discontinued. Two patients also received topotecan twice a week on days 1 and 4 and one patient originally diagnosed as PNET intrathecal methotrexate according to HIT 2000 SKK (12) (**Table 1**). Despite fatal local recurrences none of the patients developed leptomeningeal disease.

### Radiotherapy

Radiotherapy started six weeks after GTR in the two long-term survivors and 3.5 to 12 months after diagnosis in the remainder patients (**Table 1**). At the time of initiation of radiotherapy only three (cases 2,6, and 7) of the seven patients had not progressed under chemotherapy including the two long-term survivors who were irradiated early, six weeks after diagnosis.

### Response to Second Line Treatment

Three patients who received temozolomide following second look surgeries for recurrence progressed. Likewise, three patients who received an antiangiogenic metronomic therapy according to the MEMMAT strategy for their progression/relapse succumbed to their disease. One patient (case 9) received topotecan and olaparib for his second recurrence/progression. Olaparib required discontinuation only for a few days per cycle when the white blood cell count dropped below 1,000/mm3. However, he too progressed under this combination and died (**Supplementary Table S1**).

### Neuropsychological Outcome and HRQoL

Both long-term survivors were last tested 3.6 years after diagnosis (case 6 with 7.1 years and case 7 with 5.75 years). Fluid intelligence was within the average range for both patients, as most of the other domains (26, 28, 29, 32, 33, 35). Case 6 had difficulties in fine motor functions (ETMR was located in the right parietal lobe) (30, 31). Case 7 with a bifrontal tumor showed below average results for processing speed, long-term memory, working memory and emotion recognition (**Supplementary Figure S10**) (24–27).

The behavioral screening (proxy reports by mothers of the patients) resulted in close to average scores in nearly all domains for both patients.

With respect to HRQoL both patients showed average scores in most of the subscales and also in the total score.

## Discussion

ETMR is a rare highly malignant embryonal tumor of young children that emerged as a new entity comprising the former entities ETANTR, ependymoblastomas, and medulloepitheliomas based on overlapping molecular features.

We here describe a consecutive series of nine patients with ETMR treated at our institution since 2006. ETMR was confirmed molecularly in all patients with eight of the tumors harboring a *C19MC* amplification. The single tumor lacking the *C19MC* amplification displayed a *DICER1* mutation and clustered with other ETMRs (*C19MC* altered or NOS) (11). Seven had a primary GTR and two a very good second look PR and STR, respectively. All patients received immediate postoperative conventional chemotherapy followed by HDCT in three. Similarly, radiotherapy was part of the therapeutic concept and originally planned to be deferred until the end of chemotherapy. To prevent leptomeningeal seeding all but one patient (hypoxic brain injury) also received intrathecal therapy (**Table 1**). Interestingly, only the two patients who received GTR followed by early focal radiotherapy are long-term survivors and in excellent clinical condition.

Reviewing the literature in the context of their own series several authors described GTR as favourable prognostic factor (8, 17, 39, 40). Alexiou et al. analyzing 41 cases with available survival data concluded that patients receiving GTR or STR had a survival benefit compared to patients in whom only biopsy was performed (14 *vs.* 6 months) (39). While Alexiou found no statistically significant difference in the survival time between GTR and STR (14 *vs* 12 months), Mozes et al. in an analysis of seven relapsed cases stressed that there was a trend towards a somewhat faster recurrence among the four patients with STR (10.3 ± 0.85 months) *vs.* the three patients with GTR (14.0 ± 8.08 months) (17). Horwitz et al. reported on the French experience including 38 patients with ETMR (8). Thirty of these 38 were treated homogenously according to the PNET-HR protocol (41) and progression was observed in all patients with a STR during induction standard therapy (2 courses of etoposide-carboplatin) or sequential high-dose chemotherapy (melphalan/cisplatin/melphalan/cisplatin/thiotepa), highlighting the importance of a GTR. In our series both patients with biopsy and PR, respectively, demonstrated steady tumor progression as evidenced by six-weekly MRIs despite intensive chemotherapy.

Similar to the importance of GTR, multiple authors described a statistically significant overall survival (OS) benefit in ETMR with the addition of radiotherapy in uni- and multivariate analysis (8, 17, 39, 42, 43). Recently, Jaramillo et al. reported on seven patients with ETMR treated with proton therapy (42). Interestingly, three of his seven patients were alive >36 months after diagnosis.

Since only the two patients who received focal radiotherapy early on became long-term survivors in our series we reviewed the literature with regard to the role of initiation/timing and extent of radiotherapy in the treatment of ETMR published since 1990. The search yielded 228 cases published in peer reviewed journals providing clinical information. Including our nine patients 79 patients had a GTR, 61 a STR, 17 a PR or biopsy and in the remainder extent of resection was not known. Metastases were present at diagnosis in 30 (13%) and not reported in 14; 188 (79%) patients received chemotherapy including HDCT in 60, and 93 (39%) received radiotherapy as part of their treatment. Radiotherapy was performed early, delayed, at recurrence or not known in 19, 36, 20 and 18, respectively. Extent of radiotherapy was not known in 15, craniospinal (CSI) irradiation performed in 30 and focal radiotherapy in 48. Only 26 (11%) of 237 patients survived >36 months with no evidence of disease at last follow-up. ETMR was confirmed by LIN28A expression and/or *C19MC* amplification in 14 of the 26 long-term survivors (**Table 2**). Nineteen of the 26 had a GTR, four a STR and in three extent of resection was not available. All but two long-term (>36 months) survivors received radiotherapy. Radiotherapy was CSI in 10, focal in 11, and not known in three. Ten of the survivors were irradiated early on following GTR, two of whom received only radiotherapy (**Table 2**, patient numbers 1, 4, 9, 11, 12, 14, 23–26). Thirteen received radiotherapy after intensive chemotherapy including HDCT in nine and at least four (**Table 2**, patient numbers 2, 17, 19, and 22) of these 13 patients had progressed before radiotherapy. For one survivor type of therapy is not known.

**Table 2:** Characteristics of all patients with no evidence of disease >36 months.

Author	Pt. number	Age (months)	Gender	Location	Surgery	M-Stage	CT	RT	HDCT	Follow-up (months)	LIN28A	C19MC
Manjila et al. (44)	1	48	m	parietal	GTR	M0	CCG 99701	CSI	no	84	n/a	n/a
Mozes et al. (17)	2	55*	f	cerebellum	STR*	M0	TMZ*	CSI*	no*	89*	n/a	n/a
Alexiou et al. (39)	3	7	m	temporoparietal	GTR	M0	Head Start II	no	yes	48	n/a	n/a
Müller et al. (45)	4	84	f	posterior fossa	GTR	M0	HIT2000 > 4 years MB	CSI	no	82	n/a	n/a
Müller et al. (45)	5	96	f	posterior fossa	STR	M0	MET HIT 2000 AB4	CSI	no	83	n/a	n/a
Spence et al. (2)	6	23	f	cerebrum	n/a	M1	yes	yes	n/a	56	pos	amp
Spence et al. (2)	7	39	f	cerebrum	n/a	M0	yes	no	n/a	165	pos	amp
Spence et al. (2)	8	24	f	cerebrum	n/a	n/a	n/a	n/a	n/a	204	n/a	amp
Jaramillo et al. (42)	9	57	m	spine	GTR	M0	ACNS 0332	CSI	yes	44	n/a	n/a
Jaramillo et al. (42)	10	10	m	cerebellopontine	STR	M0	CCG-99703	focal	yes	36	n/a	n/a
Molloy, 1996	11	15	n/a	cerebellum	GTR	M0	i.th. MTX	CSI	no	>161	n/a	n/a
Molloy, 1996	12	17	n/a	temporoparietal	GTR	M0	VCR/VP16/cisplatin/CPM	focal	yes	>44	n/a	n/a
Gessi et al. (3)	13	36	f	frontal	resection	M0	yes	yes	n/a	42	n/a	n/a
Horwitz et al. (8)	14	138	f	supratentorial	GTR	M0	–	focal	no	183,9	pos	n/a
Horwitz et al. (8)	15	35	m	spinal cord	GTR	M3	VP-Carbo 2x	CSI	Mel/Cis2x/Thio	43,2	pos	n/a
Horwitz et al. (8)	16	26	f	supratentorial	GTR	M0	VP-Carbo/Carbo	focal	Mel/Cis2x/Thio	143,6	pos	n/a
Horwitz et al. (8)	17	38	f	supratentorial	STR	M0	VP-Carbo 2x	focal	Mel/Cis/Thio	90,9	pos	n/a
Horwitz et al. (8)	18	82	f	supratentorial	GTR	M0	VP-Carbo 2x	focal	Mel/Cis2x	63,8	pos	n/a
Horwitz et al. (8)	19	24	f	supratentorial	STR	M0	VP-Carbo 2x	focal	Mel2x/Thio2x	143,4	pos	n/a
Horwitz et al. (8)	20	31	f	supratentorial	GTR	M0	VP-Carbo 2x	focal	Mel/Cis2x/Thio	103,3	pos	n/a
Horwitz et al. (8)	21	139	f	infratentorial	GTR	M3	VP-Carbo 2x	CSI	Thio 2x/TMZ	70,5	pos	n/a
Gerber et al. (40)	22	67	m	frontotemporal	GTR	M0	HIT91 sandwich arm	CSI	no	152	n/a	n/a
Gerber et al. (40)	23	41	m	parietal	GTR	M0	HIT91 maintenance arm	CSI	no	113	n/a	n/a
Matsumoto et al. (46)	24	96	m	frontal	GTR	M0	–	focal	no	60	pos	amp
MUV case 6	25	38	f	parietal	GTR	M0	PEI 2x/TMZ	focal	no	56	pos	amp
MUV case 7	26	27	f	bifrontal	GTR	M0	PEI 2x/TMZ	focal	no	50	pos	amp

Pt. number, patient number; CT, chemotherapy; RT, radiotherapy; *, at recurrence/progression; HDCT, high-dose chemotherapy; GTR, gross total resection; STR, subtotal resection; n/a, not available; TMZ, temozolomide; MUV, Medical University of Vienna; i.th., intrathecal; CSI, craniospinal; Mel, melphalan; Cis, cisplatin; Thio, thiotepa; pos, positive; amp, amplified; * at relapse.

While GTR and radiation therapy were described as favourable prognostic factors by multiple authors, the impact of HDCT on survival is less clear. Alexiou et al. described a trend towards a better survival in patients receiving HDCT (39). Horowitz et al. reporting on the French series of 38 patients with ETMR found that with decreasing significance GTR, radiotherapy and HDCT, respectively, were associated with a better overall survival (8). Of the two only ones surviving patients who did not receive radiotherapy one received HDCT and in the other information was not available (**Table 2**).

Except for one patient who died after HDCT all patients in our series in whom radiotherapy was deferred recurred despite intensive chemotherapy and succumbed to their disease. Recently, Lambo et al. reported on the genomic landscape of ETMRs and found that relapsed tumors have a large increase in somatic single nucleotide variants (SNVs) compared to primary tumors (12). Many of these newly acquired SNVs were found to be associated with a mutational signature related to platinum-based agents, suggesting that they were induced by treatment. These findings may explain the fast evolution of resistance to chemotherapy in these patients and emphasize the importance for early use of radiotherapy. The same authors also described a prevalent genomic instability in ETMR caused by widespread occurrence of R-loop structures and demonstrated that topoisomerase 1 (TOP1) inhibitors and Poly ADP-ribose polymerase (PARP) inhibitors act synergistically in increasing the amount of DNA damage by increasing the R-loops in ETMR cells. Based on these findings we used a combination of topotecan and olaparib in one of our patients (case 9) for his second recurrence/progression. Treatment at the chosen dose was well tolerated and could be repeated every three weeks. Unfortunately, the combination treatment was not effective in this recurrent tumor. It is possible that the effect of topotecan could not be enhanced by olaparib, the only PARP inhibitor available to us at the time, because of its poor CNS penetration.

Based on the literature search including our own patients the median age of patients at presentation was 37 months (range 0 to 276), 53 (22%) were below the age of 18 months and 23 (10%) below 12 months. Only 30 of 237 (13%) were metastatic at diagnosis. This would allow for confining radiotherapy to the tumor bed in the majority of patients if given early on. Recently, encouraging results of long-term HRQoL were reported for 59 pediatric brain tumor survivors receiving proton radiotherapy at a median age of 2.5 years (range 0.3–3.8 years) (47). While the data for HRQoL outcomes were variable and significantly associated with the severity of neurologic injury at the time of diagnosis and treatment, approximately one third of the patients achieved HRQoL scores comparable to those of healthy children, and 90% of children functioned in a regular classroom. This is in accordance with the observation in our two surviving patients whose neuropsychological outcome and HRQol were within the average range in most domains 3.6 years after diagnosis. Case 6 is the best student of her class in elementary school. Concerns over negative sequelae of radiation therapy in young children may therefore be alleviated to a certain extent by an advanced radiotherapy approach such as proton therapy, in particular if only focal therapy with a markedly reduced radiation exposure to the surrounding brain is used. Taking into account area and size of the treatment volume a possible cut-off age of 18 months may be discussed. It has to be noted though that both our patients with a positive neuropsychological outcome were 38 and 27 months old at diagnosis. Nevertheless future protocols or guidelines recommending early focal radiotherapy following for example two cycles of chemotherapy may be suitable for the majority of patients given the median age of presentation for ETMRs of 37 months. Whether the concomitant use of temozolomide to radiotherapy was of additional benefit for the patients cannot be determined independently given the small numbers and the fact that two long-term survivors reported in the literature (8, 44) received focal radiotherapy only. Furthermore, while both patients receiving temozolomide chemoradiotherapy for primary diagnosis are long-term survivors this approach was not effective in two patients following tumor progression under intensive conventional chemotherapy. This is in contrast to the case report by Mozes et al. (17) who reported efficacy of concomitant temozolomide chemoradiotherapy followed by temozolomide cycles in a patient with an ETMR recurrence after intensive chemotherapy including HDCT.

Leptomeningeal spreading in patients receiving focal radiotherapy only might be prevented by intrathecal chemotherapy. In contrast to the French series (8) all our patients recurred locally and none developed leptomeningeal disease suggesting that intrathecal therapy consisting of etoposide alone or etoposide alternating with liposomal or aqueous cytarabine and/or topotecan, is effective although only topotecan is supported by preclinical evidence (48). This is in accordance with an observation by Gessi et al. (3) who described that widespread meningeal metastases resolved transiently in one patient after intrathecal therapy. While early radiotherapy might be an option for the majority of patients, infants <18 months of age, infants with a critical treatment area or volume or primary metastasized tumors will not be eligible for this approach. In these patients early intensive chemotherapy including HDCT possibly followed by maintenance therapy including novel agents might be an option.

## Conclusion

ETMR is a very rare, aggressive embryonal tumor with reported survival times averaging 12 months. Because of the median age of 37 months at presentation treatment typically consists of surgery followed by chemotherapy and delayed or no radiotherapy. Our institutional experience and a literature search suggest that GTR and early radiotherapy are potentially pivotal for long-term survival (>36 months) of patients with ETMR in the absence of effective targeted therapies.

Because only 13% of the patients presented with metastatic disease focal radiotherapy may suffice in the majority of patients if intrathecal therapy is used to prevent leptomeningeal dissemination. The use of advanced radiation technology with reduced exposure of uninvolved brain and reports of a reasonably good HRQol also of young children with proton therapy may mitigate the fear of prohibitive late effects of early focal radiotherapy in the majority of patients.

## Data Availability Statement

All data generated or analyzed for this study can be found in the article [and its **Supplementary Material**] or are publicly available within previous publications by Lambo et al. (12) for 193 ETMRs in the manuscript by Sturm et al. (37) for 534 other CNS tumors, the article by Capper et al. (13) for 44 pineoblastomas. The DNA methylation profiles generated during this study have been deposited in GEO with the accession number GSE160958.

## Ethics Statement

The studies involving human participants were reviewed and approved by Ethics Committee of the Medical University of Vienna. Written informed consent to participate in this study was provided by the participants’ legal guardian/next of kin. Written informed consent was obtained from the minor(s)’ legal guardian/next of kin for the publication of any potentially identifiable images or data included in this article.

## Author Contributions

LM provided clinical data, reviewed the literature, wrote the first draft of the manuscript, and critically reviewed the manuscript for intellectual content. JG provided input on molecular data, designed figures, and critically reviewed the manuscript for intellectual content. AP provided clinical input on interpretation of the results and critically reviewed the manuscript for intellectual content. AA provided clinical input on interpretation of the results and critically reviewed the manuscript for intellectual content. NS provided clinical input and critically reviewed the manuscript for intellectual content. TP provided data on neuropsychological outcome and health-related quality of life. TC provided clinical input on interpretation of the results and critically reviewed the manuscript for intellectual content. CD provided clinical input on interpretation of the results and critically reviewed the manuscript for intellectual content. SL performed molecular analyses and critically reviewed the manuscript for intellectual content. KD provided data on radiotherapy and critically reviewed the manuscript for intellectual content. CH provided neuropathological data and critically reviewed the manuscript for intellectual content. MK provided data on molecular analyses and critically reviewed the manuscript for intellectual content. IS initiated publication of the series, provided input on interpretation of the results, and completed the manuscript. All authors contributed to the article and approved the submitted version.

## Funding

This study was supported by the Austrian Science Fund (I 4164 to J.G.) within the TRANSCAN-2 project “BRCAddict”. SL and MK are supported by the Solving Kid's Cancer foundation and the Bibi Fund for Childhood Cancer Research.

## Conflict of Interest

The authors declare that the research was conducted in the absence of any commercial or financial relationships that could be construed as a potential conflict of interest.

## Abbreviations

ETMR, Embryonal tumors with multilayered rosettes; ETANTR, Embryonal tumor with abundant neuropil and true rosettes; CNS, Central nervous system; PNET, Primitive neuroectodermal tumor; WHO, World Health Organisation; NOS, Not otherwise specified; HDCT, High dose chemotherapy; MUV, Medical University of Vienna; GTR, Gross total resection; STR, Subtotal resection; PR, Partial resection; RANO, Response assessment in neuro-oncology; IHC, Immunohistochemical; iFISH, Interphase fluorescence in situ hybridization; FFPE, Formalin-fixed paraffin-embedded tissue; ATRT, Atypical teratoid rhabdoid tumor; IP-CZD, Polish infant protocol; Depocyte, Liposomal cytarabine; 3D, Three dimensional; GTV, Gross tumor volume; CTV, Clinical target volume; PTV, Planning target volume; VMAT, Volume mediated radiotherapy; MEMMAT, (MEMMAT;ClinicalTrials.gov Identifier: NCT01356290); BID, Bis in die (two times per day); PNET-HR protocol, Primitive neuroectodermal tumor high-risk protocol; HRQoL, Health-related quality of life; MRI, Magnetic resonance imaging; CNV, Copy number variations; OS, Overall survival; CSI, Craniospinal irradiation; SNV, Single nucleotide variants; TOP1, Topoisomerase 1; PARP, Poly (ADP-ribose) polymerase.
