# Supplementary material for: Potential Importance of Early Focal Radiotherapy Following Gross Total Resection for Long-Term Survival in Children With Embryonal Tumors With Multilayered Rosettes
Source: Front Oncol. 2020 Dec 17;10:584681. doi: 10.3389/fonc.2020.584681 (PMC7773839; doi:10.3389/fonc.2020.584681)
Supplement: Supplementary file 1 [file Table_1.docx]

| Case | Recurrence  (months after Dx) | Localisation | Reoperation | CT at recurrence | Recurrence prior to first radiotherapy | Intrathecal CT | Time from 1^st^ recurrence to death  (months) |
| --- | --- | --- | --- | --- | --- | --- | --- |
| 1 | 11 | local | GTR | TMZ, antiangiogenic metronomic therapy | yes | VP16, Depocyte | 16 |
| 2 | 10 | local | GTR | antiangiogenic metronomic therapy | - | VP16 | 4 |
| 3 | - | - | - | - | - | - | - |
| 4 | 3 | local | - | - | - | - | 12 |
| 5 | 2 | local | - | antiangiogenic metronomic therapy | yes | VP16 | 11 |
| 6 | - | - | - | - | - | - | - |
| 7 | - | - | - | - | - | - | - |
| 8 | 3 | local | PR | TMZ | yes | VP16 | 8 |
| 9 | 4 | local | STR | 1^st^ progression: TMZ, 2^nd^: Topo, olaparib | yes | VP16, Topo | 10 |

Table S1. Treatment at recurrence

Abbreviations:

Dx, diagnosis; GTR, gross total resection; PR, partial resection; STR, subtotal resection, CT, chemotherapy; TMZ, temozolomide; Topo, topotecan; VP16, etoposide; Depocyte, liposomal cytarabine

Abbreviations:

Dx, diagnosis; GTR, gross total resection; PR, partial resection; STR, subtotal resection; CT, chemotherapy; TMZ, temozolomide; Topo, topotecan; RT, radiotherapy

l
