# Supplementary material for: Potential Importance of Early Focal Radiotherapy Following Gross Total Resection for Long-Term Survival in Children With Embryonal Tumors With Multilayered Rosettes
Source: Front Oncol. 2020 Dec 17;10:584681. doi: 10.3389/fonc.2020.584681 (PMC7773839; doi:10.3389/fonc.2020.584681)
Supplement: Supplementary file 2 [file DataSheet_1.pdf]

Supplementary Figure S1. CNV and whole exome sequencing results case 1

(A)

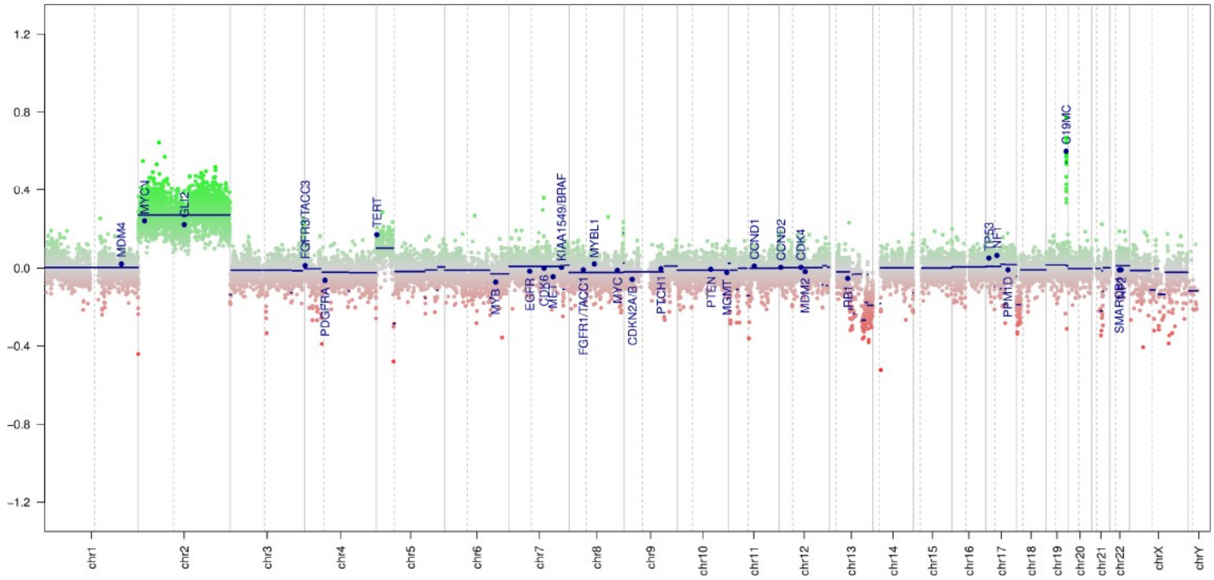

(B) Somatic mutations

No germline available.

No Dicer1 mutations detected, no rare cancer associated mutations detected (based on annotations of mutations occurring in <1% of any given population of healthy individuals).

Supplementary Figure S1. a. CNV as determined by methylation array b. somatic mutations as determined by whole exome sequencing of tumor tissue and matched germline.

Supplementary Figure S2. CNV and whole exome sequencing results case 2

(A)

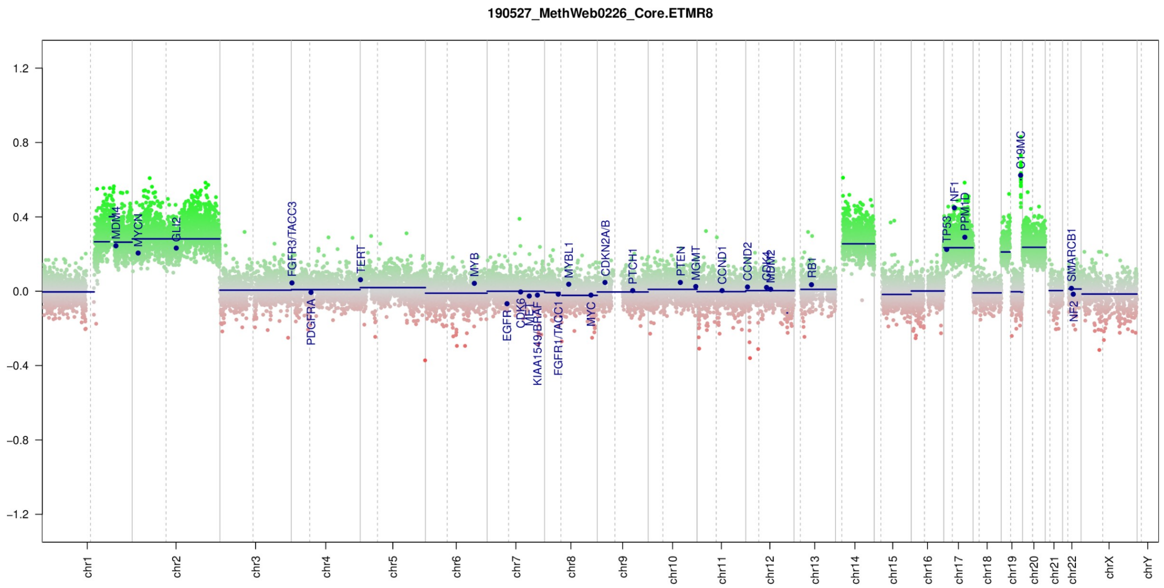

(B) Somatic mutations

Negative for Dicer1 mutations. None of the mutations have been recurrently found.

Gene	Sub	Mutation	Location	AA.Change	AF	Coverage
ABCB11	C/A	missense	c.G1108T	p.A370S	0.29	45
PLEKHG4B	G/T	missense	c.G2936T	p.R979L	0.41	41
SLC30A8	A/T	missense	c.A1020T	p.K340N	0.09	32
CACUL1	T/A	missense	c.A872T	p.Y291F	0.12	24
COL4A2	G/T	missense	c.G4130T:	p.G1377V	0.15	41
KIAA0586	C/A	missense	c.C4331A	p.P1444Q	0.70	44
RAD51	G/A	missense	c.G265A	p.A89T	0.08	37
AKAP1	G/A	missense	c.G625A	p.V209M	0.36	50
MAST3	C/G	missense	c.C3486G	p.H1162Q	0.32	22
STK35	C/G	missense	c.C1584G	p.D528E	0.38	39
BRWD3	A/C	missense	c.T760G	p.C254G	0.39	38

Supplementary Figure S2. a. CNV as determined by methylation array b. somatic mutations as determined by whole exome sequencing of tumor tissue and matched germline.

Supplementary Figure S3. CNV and whole exome sequencing results case 3

(A)

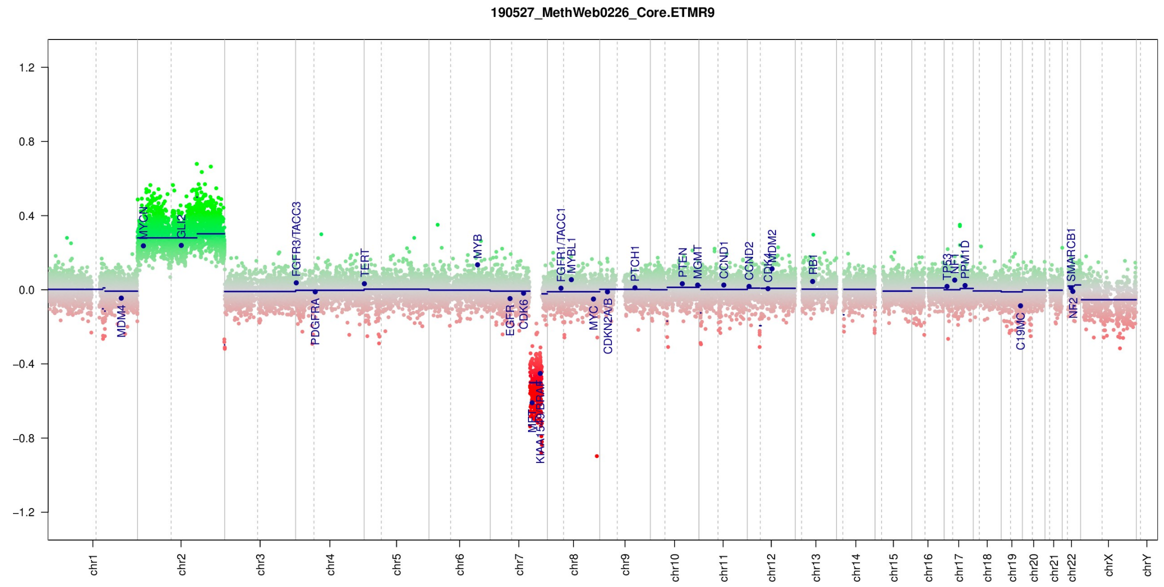

(B)

Somatic mutations

No germline available.

Does not have C19MC amplification. Otherwise CNAs are fairly typical having chromosome 2 gain, the loss on 7q is rare however. Has two hotspot Dicer1 mutations in close proximity, mutations do not co-occur on 1 read, this is indicative that both alleles are affected.

Supplementary Figure S3. a. CNV as determined by methylation array b. somatic mutations as determined by whole exome sequencing of tumor tissue and matched germline.

Supplementary Figure S4. CNV and whole exome sequencing results case 4

(A)

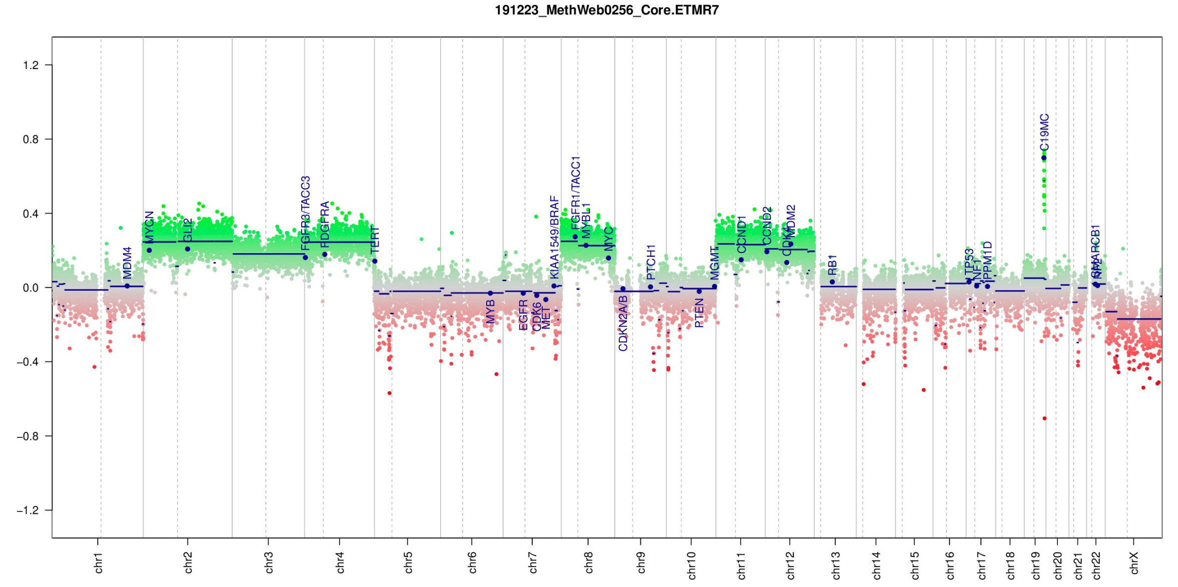

(B) Somatic mutations

No germline available.

Negative for Dicer1 mutations, no rare cancer associated mutations detected (based on annotations of mutations occurring in <1% of any given population of healthy individuals).

Supplementary Figure S4. a. CNV as determined by methylation array b. somatic mutations as determined by whole exome sequencing of tumor tissue and matched germline.

Supplementary Figure S5. CNV and whole exome sequencing results case 5

(A)

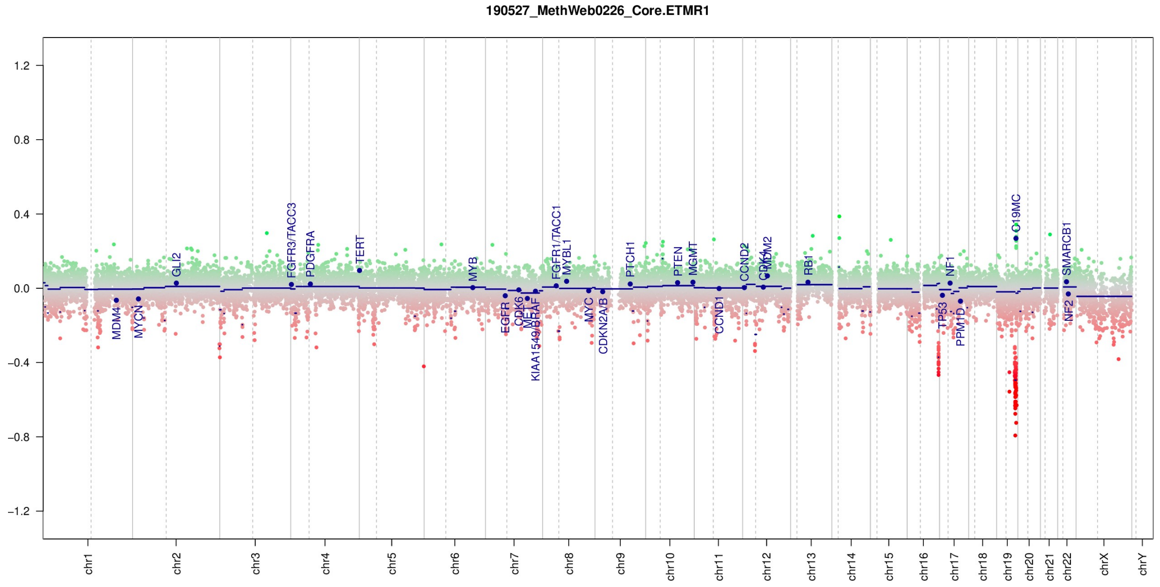

(B) Somatic mutations

Negative for Dicer1 mutations. The CHD7 mutation might be of interest.

Gene	Sub	Mutation	Location	AA.Change	AF	Coverage
CHD7	G/A	missense	c.G2215A	p.D739N	0.44	41
GDPD5	C/A	missense	c.C1528A	p.L510I	0.08	37
KLHL25	C/T	missense	c.C1034T	p.T345M	0.41	39
ATAD2	TCA/-	nonframeshift deletion	c.831_833del	p.277del	0.05	76
LGI4	CAG/-	nonframeshift deletion	c.31_33del	p.11del	0.10	39

Supplementary Figure S5. a. CNV as determined by methylation array b. somatic mutations as determined by whole exome sequencing of tumor tissue and matched germline.

Supplementary Figure S6. CNV and whole exome sequencing results case 6

(A)

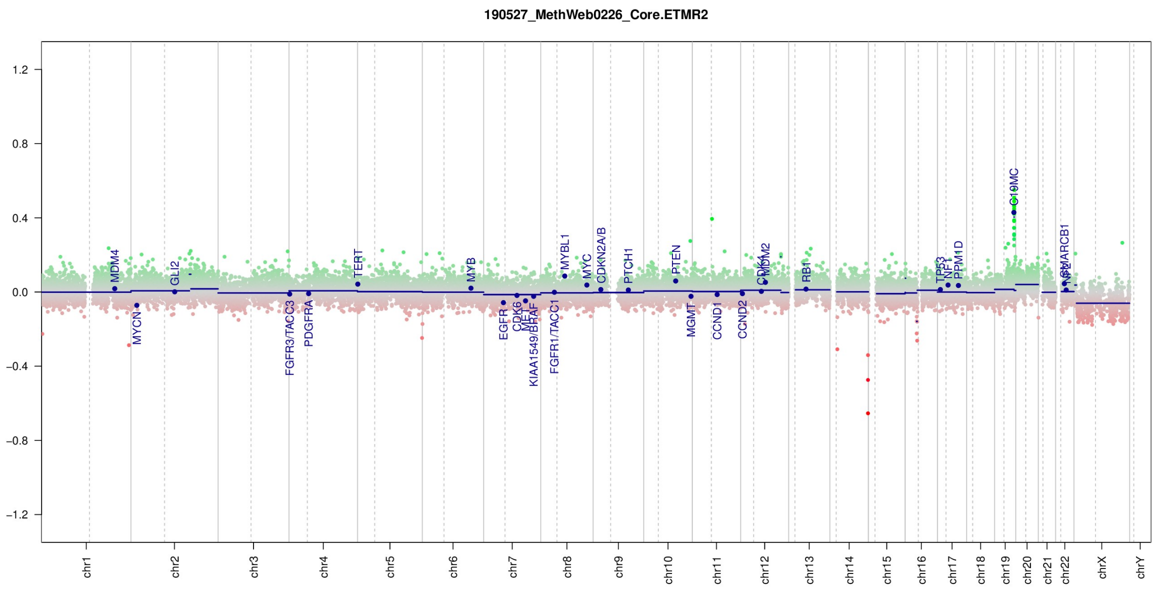

(B)

Somatic mutations

Negative for Dicer1 mutations, many SNVs/indels are likely missed.

Gene	Sub	Mutation	Location	AA.Change	AF	Coverage
USP11	C/T	missense	c.C2008T	p.P670S	0.10	48
PIK3R3	ATCTT/-	frameshift deletion	c.1057_1061del	p.K353fs	0.05	74

Supplementary Figure S6. a. CNV as determined by methylation array b. somatic mutations as determined by whole exome sequencing of tumor tissue and matched germline.

Supplementary Figure S7. CNV and whole exome sequencing results case 7

(A)

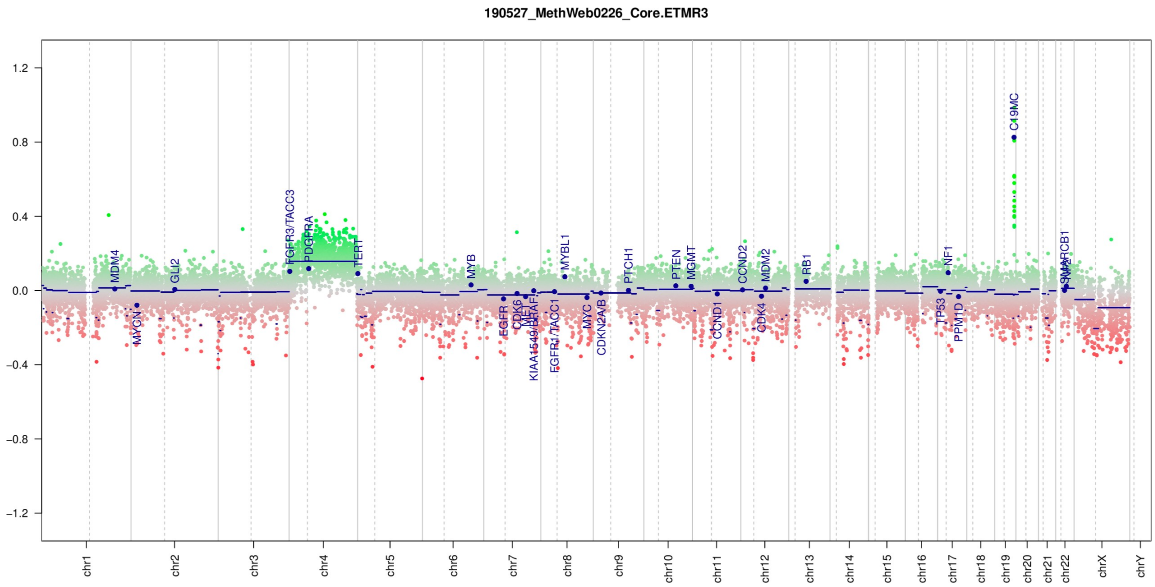

(B) Somatic mutations

Negative for Dicer1 mutations, none of the mutations have been recurrently found. The BMX mutation (STAT signalling) might be of interest.

Gene	Sub	Mutation	Location	AA.Change	AF	Coverage
MLPH	C/T	missense	c.C280T	p.R94C	0.53	34
HLA-DRB5	G/A	stopgain	c.C115T	p.Q39X	0.75	8
KIF25	A/T	missense	c.A83T	p.K28M	0.21	33
C10orf90	C/T	missense	c.G547A	p.G183S	0.12	42
SLC27A1	C/T	missense	c.C842T	p.A281V	0.57	37
WWC3	G/A	missense	c.G589A	p.V197M	0.57	35
BMX	T/A	stopgain	c.T1076A	p.L359X	0.52	40

Supplementary Figure S7. a. CNV as determined by methylation array b. somatic mutations as determined by whole exome sequencing of tumor tissue and matched germline.

Supplementary Figure S8. CNV and whole exome sequencing results case 8

(A)

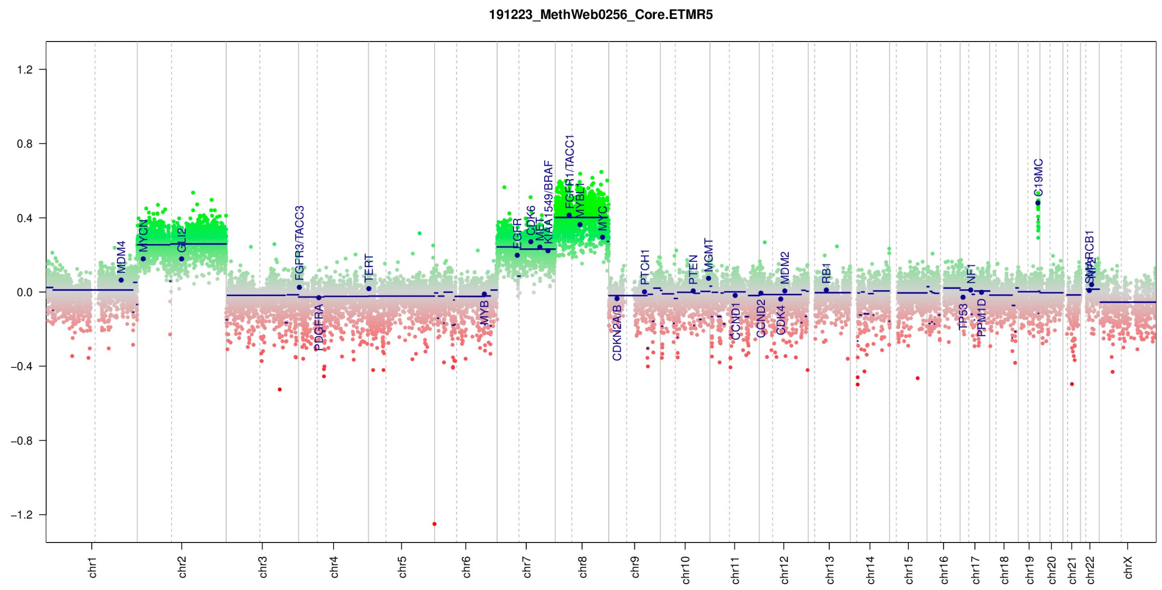

(B) Somatic mutations

Negative for Dicer1 mutations, none of the mutations have been recurrently found.

Gene	Sub	Mutation	Location	AA.Change	AF	Coverage
MAST2	G/A	missense	c.G1100A	p.R367Q	0.42	38
GPR135	G/A	missense	c.C934T	p.R312W	0.11	28
FUK	G/A	missense	c.G416A	p.G139D	0.11	27
WNK4	A/C	missense	c.A2546C	p.Q849P	0.15	27
ADAMTS10	G/A	missense	c.C173T	p.P58L	0.15	26
BCL11B	CTC/-	nonframeshift deletion	c.1411_1413del	p.471del	0.11	27

Supplementary Figure S8. a. CNV as determined by methylation array b. somatic mutations as determined by whole exome sequencing of tumor tissue and matched germline.

Supplementary Figure S9. CNV and whole exome sequencing results case 9

(A)

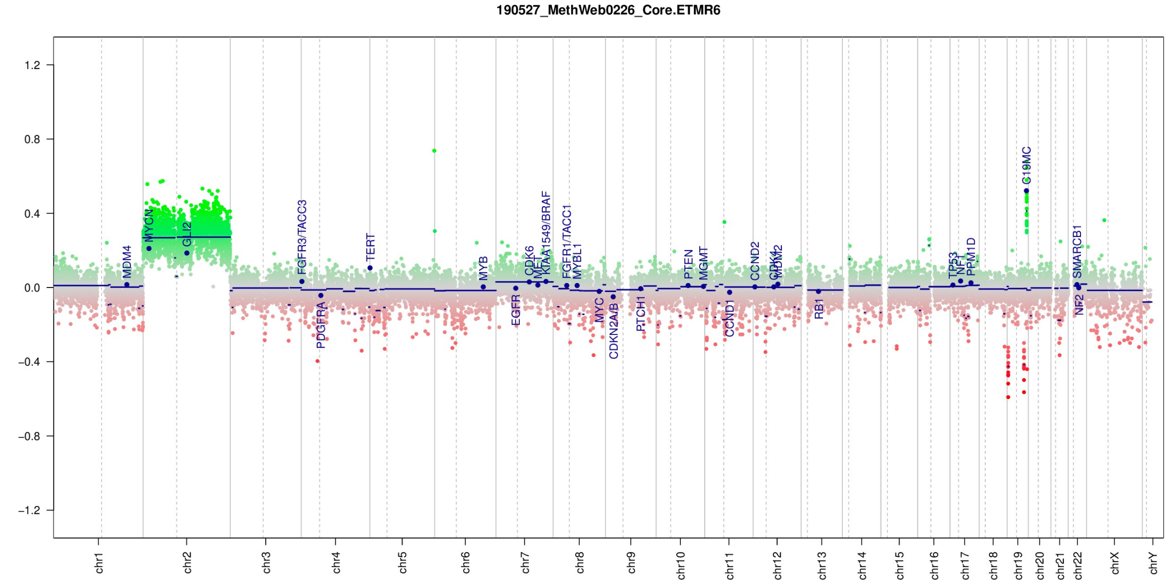

(B) Somatic mutations

Negative for Dicer1 mutations, none of the mutations have been recurrently found. The KDM5C mutation may be of interest.

Gene	Sub	Mutation	Location	AA.Change	AF	Coverage
TTN	C/T	missense	c.G6106A	p.E2036K	0.29	42
ITPR1	A/G	missense	c.A7834G	p.I2612V	0.20	30
GAK	G/A	missense	c.C3190T	p.R1064W	0.50	26
RGS12	G/A	missense	c.G29A	p.R10H	0.30	27
DTHD1	G/A	missense	c.G190A	p.V64M	0.15	27
HLA-DQA2	T/C	missense	c.T362C	p.F121S	0.13	38
SGK223	C/T	missense	c.G751A	p.G251R	0.29	35
ZHX1	G/A	missense	c.C2251T	p.R751W	0.12	24
GRIN2B	T/A	missense	c.A4051T	p.N1351Y	0.15	26
MYH6	A/C	missense	c.T3259G	p.F1087V	0.19	21
ZC3H18	C/T	missense	c.C2654T	p.P885L	0.14	28
SSC5D	C/T	missense	c.C2122T	p.R708W	0.16	44
RRBP1	G/A	missense	c.C1348T	p.R450C	0.11	28
KDM5C	G/A	missense	c.C2047T	p.R683W	0.54	24

Supplementary Figure S9. a. CNV as determined by methylation array b. somatic mutations as determined by whole exome sequencing of tumor tissue and matched germline.

Supplementary Figure S10. Neuropsychological profile of case 6 and 7

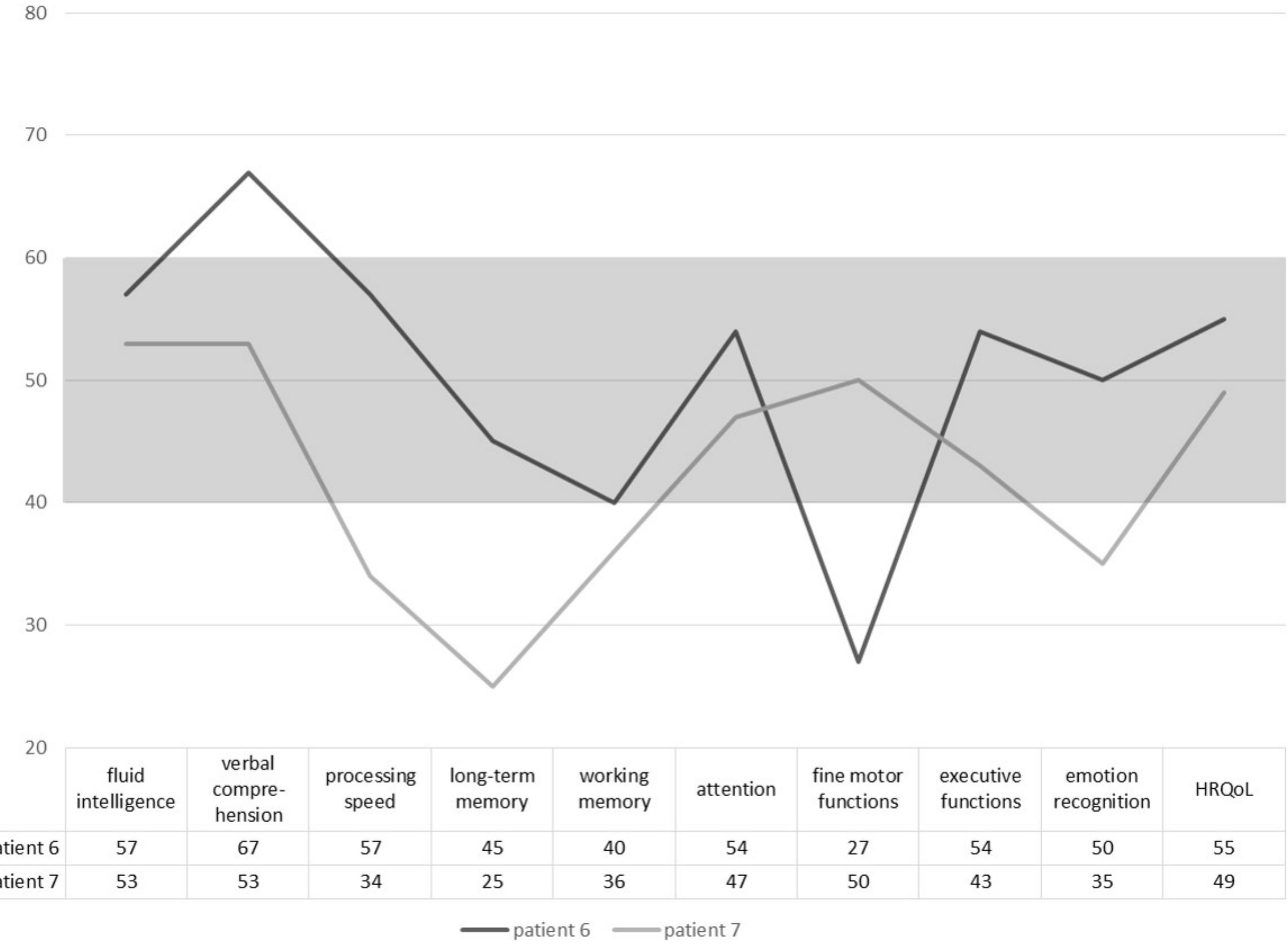

	fluid intelligence	verbal compre-hension	processing speed	long-term memory	working memory	attention	fine motor functions	executive functions	emotion recognition	HRQoL
patient 6	57	67	57	45	40	54	27	54	50	55
patient 7	53	53	34	25	36	47	50	43	35	49

Supplementary Figure S10. Neuropsychological profile of case 6 and 7; results are displayed in T-scores (mean = 50, SD = 10); the average range (+/- 1 SD) is shaded.
